# Multifaceted roles of CCL20 (C-C motif chemokine ligand 20): mechanisms and communication networks in breast cancer progression

**DOI:** 10.1080/21655979.2021.1974765

**Published:** 2021-09-27

**Authors:** Louis Boafo Kwantwi, Shujing Wang, Youjing Sheng, Qiang Wu

**Affiliations:** a Department of Pathology, School of Basic Medical Science, Anhui Medical University, Hefei, PR China; b Department of Immunology, School of Basic Medical Science, Anhui Medical University, Hefei, PR China; c Department of Pathology, The First Affiliated Hospital of Anhui Medical University, Hefei, PR China

**Keywords:** CCL20, migration, angiogenesis, chemoresistance, immunosuppression, breast cancer

## Abstract

Emerging studies have demonstrated notable roles of CCL20 in breast cancer progression. Based on these findings, CCL20 has become a potential therapeutic target for cancer immunotherapy. Accordingly, studies utilizing monoclonal antibodies to target CCL20 are currently being experimented. However, the existence of cytokine network in the tumor microenvironment collectively regulates tumor progression. Hence, a deeper understanding of the role of CCL20 and the underlying signaling pathways regulating the functions of CCL20 may provide a novel strategy for therapeutic interventions. This review provides the current knowledge on how CCL20 interacts with breast cancer cells to influence tumor progression via immunosuppression, angiogenesis, epithelial to mesenchymal transition, migration/invasion and chemoresistance. As a possible candidate biomarker, we also reviewed signal pathways and other factors in the tumor microenvironment regulating the tumor-promoting functions of CCL20.

These new insights may be useful to design new potent and selective CCL20 inhibitors against breast cancer in the future.

## Graphical Abstract

**Figure:**
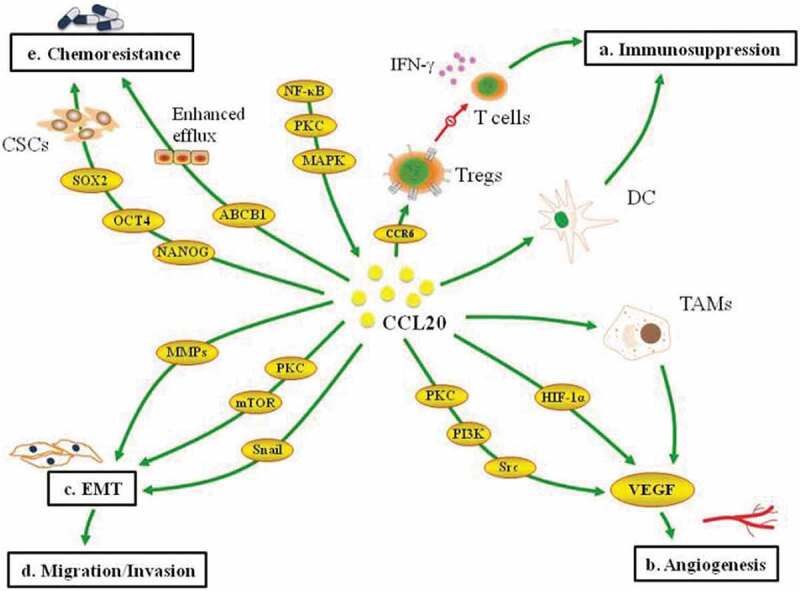

## 1. Introduction

Breast cancer continues to be the leading cause of cancer-related deaths among women [1,2], primarily as a result of metastasis. Conventionally, breast cancer metastasis is mediated by the interaction between chemokine receptors on cancer cells and chemokines expressed at the metastasis site [3]. Chemokines are low molecular weight (8–14 kDa) cytokines that are classified as CC, CXC, CX3C and C chemokines [4]. Chemokines are well known to play essential roles in the recruitment of immune cells and the development of lymphoid tissues. They also regulate the recruitment and trafficking of leucocytes during homeostasis and inflammation [5–7]. Inflammatory chemokines are released to induce leukocyte infiltration to inflammatory site [8].On the other hand, homeostatic chemokines are expressed in tissues and play essential roles in adaptive immunity [9]. Beyond these physiological roles, emerging studies have highlighted the indispensable role of chemokines and their receptors in tumor progression [7,10,11]. It has become evident that chemokines regulate several oncogenic processes, including host immune response, tumor growth, angiogenesis, metastasis and chemoresistance [3,12].

CCL20, the only chemokine ligand for C-C motif chemokine ligand-receptor 6(CCR6), is a member of the CC family and the alpha subfamily chemokines [4,13–15]. Structurally, CCL20 contains four exons and three introns at their junctions which differ from other members of the CC chemokines [16]. The available evidence supports the role of CCL20 in the progression of inflammatory diseases [17,18], including cancers [19–23]. These advances have led to the recognition of CCL20 as a promising candidate for cancer immunotherapy. Therefore, an in-depth understanding of the roles and regulatory mechanisms of CCL20 are relevant for the development of cancer drugs with improved efficacies. This review summarized the emerging roles of CCL20 and the underlying signaling regulating the tumor-promoting functions of CCL20 in breast cancer. The synergistic effect of CCL20 and other factors in the tumor microenvironment have also been discussed.

## 2. Role of CCL20 in breast cancer progression

It is well established that tumor cells express high levels of chemokines. Chemokines recruit and signal immune cells to create a favorable environment for cancer cells [24]. Previously, CCL20 was known for its antimicrobial activity [25] and involvement in autoimmune diseases such as inflammatory bowel disease [26], rheumatoid arthritis [27] and psoriasis [28]. However, emerging evidence strongly suggests association between CCL20 and tumor progression in several solid tumors. Importantly, several established roles, including tumor growth, inhibition of apoptosis, angiogenesis and therapeutic resistance have been linked to the expression of CCL20 in the tumor microenvironment [29,30]. In the remaining section, the roles of CCL20 in immunosuppression, angiogenesis, epithelial to mesenchymal transition (EMT), migration/invasion and chemoresistance have been discussed (Table 1).

**Table 1.** Summary of the role of CCL20 in breast cancer progression

Mechanism	Function	Reference
Immunosuppression	CCL20 via CCR6+ Tregs decreased the expression of IFN-γ secreted by CD8 + T cells. CCL20 recruited immature dendritic cells into tumor tissues to impair immune response.	[42,46]
EMT	CCL20 upregulated vimentin and N-cadherin but downregulated E-cadherin and ZO-1.	[23]
Migration and Invasion	CCL20 promoted the migration and invasion of triple-negative breast cancer cells. Silencing CCL20 by intraperitoneal injection of anti-CCL20 antibody inhibited osteolytic bone metastasis in mice inoculated with MDA-MB-231 cells.	[86, 87, 87]
Angiogenesis	Recombinant human CCL20 induced VEGF expression.	[23]
Tumor growth	Recombinant human CCL20 increased the proliferation of breast cancer cells (MDA-MB-231 and SUM159 cells) . Expression of CCL20 in MDA-MB-468 cell lines recruited macrophages into tumors to promote their growth.	[57,87]
Chemoresistance	CCL20 promotes renewal and maintenance of cancer stem cells in triple-negative breast cancer cells. CCL20 upregulated ABCB1 to promote chemoresistance to taxanes.	[92]

### 2.1. Immunosuppression

The ability of cancer cells to escape from the host immune response is a hallmark of cancer progression. In these processes, immune effector cells with anti-tumor functions acquire protumor functions [31,32]. Accumulating studies have shown that forkhead/ winged-helix transcription factor P3 (FOXP3) infiltrated into tumor tissues plays vital role in tumor immunity. Their presence impairs anti-tumor immune response of the host and promotes tumor progression in several solid tumors including breast cancer [33–35]. Furthermore, the number of tumor-infiltrating FOXP3+ Treg in tumor tissue increases with tumor progression [36]. Evidence has shown that Tregs are induced to invade tumor tissues by CCL20. Jinlin et al. has demonstrated that CCL20 promotes regulatory T cells (Tregs) recruitment and cancer progression in mice implanted with colorectal cancer cells [37]. According to Kang et al., marked infiltration of Tregs induced by CCL20 potentiates disease progression in hepatocellular carcinoma [38]. In a recent study, high expression of CCL20 was associated with an increased homing of FOXP3 into breast cancer tissues. The study further noted that the concomitant expressions of these markers were associated with poor prognostic outcomes in breast cancer patients [39]. Besides the chemotactic functions, CCL20 cooperates with CCR6 to suppress the functions of CD8 T cells. It is well known that the integral role played by CD8 T cells in antitumor immunity is mainly dependent on the secretion of cytotoxic molecules or effector cytokines [40]. Unsurprisingly, low levels of these effector cytokines on CD8 + T cells have been associated with immunosuppression [41]. Along this line, a study has noted that high infiltration of CCR6 Tregs promotes immunosuppression and disease progression in breast cancer by suppressing IFN-γ on CD8 + T cells [42].

It has been reported that dendritic cells infiltrated into tumors perform opposing functions depending on their maturation stage [43]. Immature dendritic cells (iDCs) present antigens to T cells, and further induce immune tolerance, including generation of inducible Tregs cells and anergy of T cells. On the other hand, mature dendritic cells prime CD4 and CD8 T cells, activate B cells and initiate the activation of an adaptive immune response [44]. Beyond recruiting Tregs, CCL20 also fosters immunosuppression through the recruitment of dendritic cells. According to Bell et al., high expression of CCL20 was associated with increased infiltration of immature dendritic cells into breast cancer tissues [45]. Dendritic cell infiltrated into tumor tissues is associated with poor prognostic outcomes in breast cancer patients [46]. According to a mechanistic study, tumor derived CCL20 induces the infiltration of immature dendritic cells with two distinct phenotypes that differ in their capacity to activate T lymphocytes [47]. However, the mechanism of reprogramming dendritic cells by CCL20 remains unknown and warrants further studies in breast cancer.

### 2.2. Angiogenesis

Like normal cells, adequate oxygen supply to tumor cells is critical for their survival [48,49]. CCL20 plays important role in angiogenesis and vascular remodeling [50,51] by stimulating angiogenic factors such as vascular endothelial growth factor (VEGF). VEGF, a crucial driver of angiogenesis, enhances tumor vessel dilation, permeability and leaking [52]. In principle, the action of VEGF is potentiated by the hypoxic nature of the tumor microenvironment, where the oxygen deficit stimulates the expression of hypoxia-inducible factor-alpha 1(HIF-1α) [53,54]. Emerging evidence shows that the treatment of healthy breast cells with recombinant human CCL20 upregulates the protein and mRNA expression of VEGF [23]. In aortic ring assay, a robust method for determining angiogenesis [55], CCL20 directly induced angiogenic microvessel aortic sprouting in primary breast cancer cells [23]. In other experimental settings, CCL20 promoted angiogenesis [51] by inducing endothelial cell proliferation and the formation of new blood vessels [56]. These outcomes were consistent in a study where the neutralization of CCL20 inhibited angiogenesis in hepatocellular carcinoma [51].

Immune cells have been identified to play essential roles in angiogenesis. Among them, a link between CCL20 and tumor-associated macrophages (TAMs) has been established. As chemokine, CCL20 plays a substantial role in the recruitment of several immune cells, including macrophages. In a recent study, silencing CCL20 in tumor conditioned media from MDA-MB-231 cells attenuated the recruitment of macrophages into tumor-bearing mice [57]. Although the authors did not provide a regulatory mechanism of CCL20 induced TAMs in angiogenesis, TAMs have been demonstrated to promote angiogenesis by supplying tumor cells with angiogenic factors [58].

### 2.3. Epithelial-mesenchymal transition (EMT)

In certain key biological processes such as tissue remodeling, embryonic development, wound repair, and tumorigenesis, epithelial cells with rigid structures acquire a flexible phenotype that allows cell migration and movement. This morphogenetic transformation is known as epithelial-mesenchymal transition [59]. Experimentally, EMT is marked by loss of E-cadherin expression (epithelial phenotype) and increased expression of vimentin and N-cadherin (mesenchymal phenotype) [60,61]. EMT is known to play important role in the oncogenesis processes by enhancing the ability of tumor cells to access blood and migrate to establish metastases [62–64]. Significantly, the induction of EMT is associated with tumor aggressiveness and poor prognostic outcomes in breast cancer patients [65,66]. The role of chemokines in EMT induction in epithelial tumor cells has been investigated [67,68], and the evidence has implicated CCL20 in such processes [69]. In vitro study has demonstrated that direct coculture between healthy breast cells and recombinant human CCL20 upregulates vimentin and N-cadherin, which are characteristics of EMT. To further establish the involvement of CCL20 in EMT, blocking CCL20 decreased the expression of vimentin and N-cadherin [23]. Besides the aforementioned EMT markers, EMT inducers can also enhance the expression of metalloproteinases (MMPs) [70]. Generally, MMPs degrade the extracellular matrix and basement membrane [71–73] to facilitate tumor metastasis [74,75]. According to Marsigliante et al., induction of EMT by CCL20 is associated with high expression of MMP-2 and MMP-9 in breast cancer cells [23].

The process of EMT is characterized by the activation of several key transcriptional factors, including Twist, Snail (SNAI1), Slug (SNAI2), zinc finger E-box-binding (ZEB) and basic helix-loop-helix (bHLH) [76–78]. These EMT transcription factors reconstruct a favorable tumor microenvironment to facilitate immune evasion [79]. It has been demonstrated that blockade of Snail in healthy breast cells treated with CCL20 restores the expression of E-cadherin and zonula occludens-1(ZO-1) but downregulates that of N-cadherin and vimentin [23], suggesting the involvement of Snail in CCL20 driven EMT.

### 2.4. Migration and invasion

The migration of tumor cells from their primary organ is essential for the metastatic dissemination of tumors. This process largely depends on the biochemical and physical properties of cells and the extracellular matrix [80]. Chemokines and their receptors directly affect the invasive abilities of tumor cells by binding to the glycosaminoglycans in the extracellular matrix [81]. In several solid tumors, CCL20 has been identified to potentiate the migration and invasion of tumor cells [82–85]. Using adipocyte conditioned media (ACM) in coculture with MDA-MB-231 cells, Kim et al. demonstrated a higher invasive ability of MDA-MB-231 cells than MDA-MB-231 cells cultured alone. The study further noted that migration of MDA-MB-231 cells was associated with increased production of CCL20 in adipocyte-conditioned media [86]. Lee et al. investigated the effect of chemokines on the migratory and invasive abilities of breast cancer cells and found CCL20 to play key role in such processes. More importantly, silencing CCL20 by intraperitoneal injection of anti-CCL20 antibody inhibited osteolytic bone metastasis in mice inoculated with MDA-MB-231 cells [87].

Degradation of extracellular matrix by proteinase such as MMPs has been shown to play pivotal roles in tumor invasion [88,89].To gain insights into CCL20 mediated invasion mechanisms, Lee et al. determined the expression of MMPs in triple-negative and luminal breast cancer cell lines. The expression of CCL20 in triple-negative breast cancer cell lines (MDA-MB-231 and BT549) facilitated tumor migration by upregulating MMP1, MMP-2 and MMP-9. On the contrary, CCL20 in MCF-7 and ZR-75-1 cell lines showed no significant effect on tumor invasion and expression of MMPs [87]. More interestingly, these observations have been noted in gastric [90] and thyroid [91] cancers where CCL20 enhanced the migration and invasion of tumor cells through MMPs. These data strongly suggest that CCL20, through MMPs, exerts more significant effects in metastatic triple-negative breast cancer cells [92] than less invasive luminal breast cancer cells.

### 2.5. Chemoresistance

During cancer treatment, surviving cells reemerge as more aggressive cells to limit further treatment options. This phenomenon has been a challenge in cancer treatment and patient survival [93]. Drug resistance has been linked to increased immunosuppressive cells, chemokines, and cytokines [94–96]. Changes in the levels of cytokines during chemoresistance have been reported to favor tumor growth and disease progression [97]. Evidence implicating chemokines in drug resistance has been obtained through serum evaluation of chemokines and mechanistic studies(in vivo and in vitro) [98]. According to Goulin et al., high serum levels of chemokine (C-X-C motif) ligand 13 (CXCL-13) in colorectal patients are associated with 5-fluorouracil drug resistance [99]. Chen et al. also evaluated serum levels of CCL20 before, during and after treatment of taxanes in breast cancer. The study found high serum levels of CCL20 during and after taxanes treatment, especially in patients with non-pathologic complete response [92]. Emerging evidence supports a link between cancer stem cells renewal and chemotherapeutic resistance [100,101]. These processes alter genes controlling cell cycle and apoptosis [102]. Chen et al. have shown that CCL20 fosters the resistance of breast cells to taxanes by upregulating stem cell genes such as NANOG, octamer-binding transcription factor 4 (OCT4), and sex-determining region Y-box 2 (SOX2) [92]. In another experimental study, CCL20 increased the expression of OCT4 to promote resistance of ovarian cancer cells to paclitaxel [103]. Besides the upregulation of cancer stem cell genes, enhanced efflux of drugs plays crucial role in therapeutic resistance [104]. Largely, drug efflux is potentiated by the expression of transmembrane transporters, including ATP-binding cassette subfamily B member 1(ABCB1) [104]. Chen et al. investigated the association between CCL20 and ABCB1. The researchers found that overexpression of CCL20 in triple-negative breast cancer cell lines induced high expression of ABCB1, which further promoted taxanes resistance [92]. Collectively, targeting CCL20 could promote chemotherapeutic efficacy in breast cancer.

## 3. Signal pathways regulating CCL20 in breast cancer

**Figure 1.**
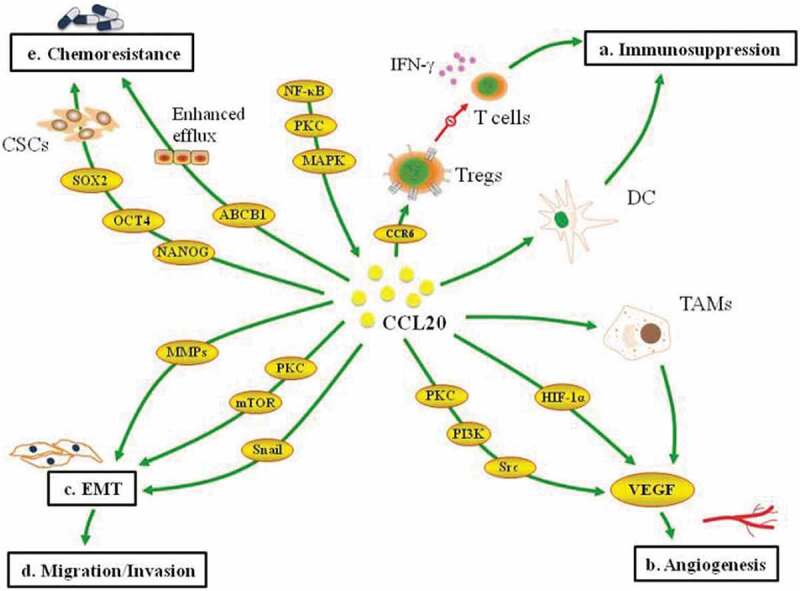
Multifaceted roles of CCL20 in breast cancer progression. CCL20 recruits dendritic cells and CCR6 Tregs to impair the function of T cells (a). CCL20 induces VEGF expression to foster angiogenesis (b). CCL20 activates Snail to upregulates mesenchymal markers and downregulates epithelial markers (c).CCL20 promotes the migration and invasion of cancer cells. CCL20 induces stem cell genes and ABCBI expression to enhance drug resistance (e)

The signal pathways regulating the actions of CCL20 in breast cancer are diverse and complex, where the activation of a pathway signals several other downstream pathways. Besides the well-known functions of nuclear factor-kB (NF-kB) in regulating immune response and inflammation [105,106], increasing evidence supports their involvement in several oncogenic processes [105,107]. They are known to regulate the expression of proinflammatory genes, including those encoding chemokines and cytokines [106]. In tipple negative breast cancer cell lines, silencing NF-kB reduced the expression of stem cell genes and ABCB1. Consequently, taxanes resistance induced by CCL20 was reversed. Notably, these activities of NF-kB were regulated through protein kinase c (PKC) and mitogen-activated protein kinase (MAPK) signaling pathways [92]. Kim et al. showed in triple-negative breast cancer cell lines that the inhibition of NF-kB downregulates the expression of CCL20 in adipocyte conditioned media [86]. In a related study, the inhibition of NF-kB, PKC and mammalian target of rapamycin (mTOR) downregulated the expression of vimentin and N-cadherin induced by CCL20, suggesting the involvement of these kinases in CCL20-driven EMT [23]. Evidence provided by Marsigliante et al. showed that PKC, Src and phosphoinositide 3-kinase (PI3K) are key regulators of CCL20-driven angiogenesis in breast cancer (Figure 1) [23].

Notch signaling controls several cellular processes, such as cell-fate specialization, maintenance of stem cells, motility, proliferation and survival. However, the abnormal expression of Notch influences the oncogenic processes in breast cancer through these activities [108]. Notch signaling regulates oncogenic activities [109] and controls the expression of several target genes, including CCL20 [110]. In a recent study exploring downstream regulators of notch signaling in the metastasis processes of breast cancer, silencing notch in MDA-MB-231 cell lines significantly inhibited the mRNA expression of CCL20 [111]. Although this study did not establish any tumor-promoting effect of CCL20 via notch signaling, the independent role of notch and CCL20 makes it logical to assume that CCL20 may influence tumor progression via notch pathway in breast cancer.

## 4. Communication between CCL20 and other factors in breast cancer tumor microenvironment

Beyond the direct role of CCL20 in influencing tumor progression, several studies have highlighted the synergistic effect of CCL20 and other cytokines in promoting tumor progression. In colorectal cancer, CCL20 in collaboration with C-X-C motif chemokine ligand (CXCL8), promoted tumor metastasis through EMT [69]. In non -small-cell-lung-cancer (NSCLC), interleukin 17 (IL-17) induced the expression of CCL20, and their concomitant expression promoted the disease progression [112].In breast cancer, evidence has shown that the tumor-promoting functions of CCL20 can be regulated by human antigen R (HuR), Cbp/P300 interacting transactivator with Glu/Asp-rich carboxyl-terminal domain 2(CITED2) and tumor necrosis factor-alpha (TNF-α) (Table 2).

**Table 2.** Signaling or factors regulating the functions of CCL20 in breast cancer

Factor	Function	Reference
Notch	Silencing notch in MDA-MB-231 cell lines significantly downregulated CCL20.	[111]
NF-kB	NF-kB inhibition in MDA-MB-231 cell lines reduced the expression of CCL20 induced by adipocyte conditioned media. Blockade of p65NF-kB in TNBC cell lines downregulated the expression of ABCB1 and reversed taxanes resistance induced by CCL20.	[86,92]
NF-kB, PKC and mTOR	Activation of NF-kB, PKC and mTOR in breast cancer cells regulated the expression of vimentin and N-cadherin induced by CCL20.	[23]
PKC, Src and PI3K	Activation of PKC, Src and PI3K regulates the expression of VEGF mediated by CCL20.	[23]
HuR	HuR regulates the expression of CCL20 in breast cancer cells	[87]
CITED2	CITED2 regulated the recruitment of macrophages induced CCL20.	[57]
TNF-α	TNF-α regulates the expression of CCL20 in TNBC.	[86]

Human antigen R (HuR), a member of the embryonic lethal abnormal vision, binds to the 3′ untranslated regions of the target mRNA to enhance their stability. Overexpression of HuR has been reported to modulate the oncogenic processes by upregulating the expression of growth factors [113,114]. In breast cancer, HuR regulated the expression of CCL20 to promote invasion of MDA-MB-231 cells and osteolytic bone metastasis in tumor-bearing mice. Furthermore, silencing either CCL20 or HuR in triple-negative breast cell lines significantly abrogated the tumor-promoting effect. These suggest that although HuR can upregulate the expression of CCL20, they exhibit a synergetic effect on tumor behavior [87].

CITED2 is a transcription co-factor that plays vital role in various fundamental cellular processes during development and differentiation. However, mounting evidence suggests a potential role of CITED2 in the development and progression of several human malignancies, including breast cancer [115,116]. According to previous studies, CITED2 regulates the expression of several growth factors such as transforming growth factor-beta (TGFβ) [115], interleukin 11 (IL-11), interleukin 1 beta (IL-1β) [117] and HIF-1α [118] to promote breast cancer progression. In breast cancer, the regulatory effect of CITED2 on CCL20 has been reported by Jayaraman et al. According to the authors, silencing CITED2 in MDA-MB-231 cell line attenuates the recruitment of macrophages mediated by CCL20 [57].

TNF-α is a crucial inflammatory cytokine in the tumor microenvironment that plays active role in all stages of breast cancer progression [119]. TNF-α regulates the expression of CCL20 in various pathological conditions such as autoimmune hepatitis [120] and psoriasis [28].In breast cancer, Kim et al. investigated the factor responsible for upregulating the expression of CCL20 in MDA-MB-231 cell line. The authors showed that silencing TNF-α using TNF-α neutralizing antibody abolished the expression of CCL20 induced by adipocyte conditioned media [86].

Despite the limited evidence in this area, a better understanding of the communication between CCL20 and other factors in the tumor microenvironment will provide insights into tumor biology. This will be beneficial in developing a dual-antagonist combination treatment for breast cancer.

## 5. Conclusion

Collectively, this review suggests that CCL20 plays essential roles in the oncogenesis processes of breast cancer. However, CCL20 appears to engage in a complex interaction with other factors in the tumor microenvironment. Hence, as research advances, it would be better to understand the communication networks between CCL20 and other factors in the tumor microenvironment. Notably, there is the need to highlight and understand the key factors regulating the tumor-promoting effects of CCL20 in tumor progression. As we gain such new insights, disrupting CCL20 promoting networks could provide novel therapeutic strategies for breast cancer treatment.

## Article Highlights

1. The roles of CCL20 in breast cancer progression have been summarized.
2. Communication between CCL20 and other factors in breast cancer tumor microenvironment have been reported.
3. Targeting CCL20 can be a useful strategy for breast cancer treatment.
